# Supplementary material for: From sequence to enzyme mechanism using multi-label machine learning
Source: BMC Bioinformatics. 2014 May 19;15:150. doi: 10.1186/1471-2105-15-150 (PMC4229970; doi:10.1186/1471-2105-15-150)
Supplement: Additional file 2 — Java code of ml2db. Additional file ml2db_code.tar.gz contains the Java source code to run the multi-label machine learning experiments and save the results to database. The code’s Javadoc is included. [file 1471-2105-15-150-S2.zip › additional file 2/ml2db/utils/doc/uk/ac/standrews/utils/main/maputils/class-use/OneToManyMap.html]

Uses of Class uk.ac.standrews.utils.main.maputils.OneToManyMap


JavaScript is disabled on your browser.


- Overview
- Package
- Class
- Use
- Tree
- Deprecated
- Index
- Help

- Prev
- Next

- Frames
- No Frames

- All Classes

## Uses of Class uk.ac.standrews.utils.main.maputils.OneToManyMap

- Packages that use OneToManyMap

  | Package | Description |
  | --- | --- |
  |  |  |
  | --- | --- |
  | uk.ac.standrews.utils.main.collectionutils.setutils |  |
  | uk.ac.standrews.utils.main.maputils |  |
  | uk.ac.standrews.utils.main.setutils |  |
  | uk.ac.standrews.utils.test.maputils |  |
- - ### Uses of OneToManyMap in uk.ac.standrews.utils.main.collectionutils.setutils

    Methods in uk.ac.standrews.utils.main.collectionutils.setutils that return OneToManyMap

    | Modifier and Type | Method and Description |
    | --- | --- |
    |  |  |
    | --- | --- |
    | `OneToManyMap` | SupersetsManager.`getSupersetsIds()` |
  - ### Uses of OneToManyMap in uk.ac.standrews.utils.main.maputils

    Methods in uk.ac.standrews.utils.main.maputils that return OneToManyMap

    | Modifier and Type | Method and Description |
    | --- | --- |
    |  |  |
    | --- | --- |
    | `static <T> OneToManyMap<T,T>` | MapUtils.`fromArrayOfArrayToOneToManyMap(T[][] array)` Array format must be [[key1, value1][key1, value2],[key2, value3],[...]]. |
    | `OneToManyMap<java.lang.String,java.lang.String>` | MapOfMaps.`getExternalInternalKeyMap()` A one to many map containing for each external key the list of unique internal keys in its map. |
    | `OneToManyMap<java.lang.String,java.lang.String>` | TableMap.`getMap()` Get the keys to values map |
    | `OneToManyMap<java.lang.String,java.lang.String>` | TableMap.`getReverseMap()` Get the values to keys map |
    | `static OneToManyMap<java.lang.String,java.lang.String>` | OneToManyMap.`invertKeysAndValues(OneToManyMap<java.lang.String,java.lang.String> map)` |
    | `OneToManyMap<T,U>` | OneToManyMap.`removeEmptyStrings()` |

    Methods in uk.ac.standrews.utils.main.maputils with parameters of type OneToManyMap

    | Modifier and Type | Method and Description |
    | --- | --- |
    |  |  |
    | --- | --- |
    | `void` | OneToManyMap.`addAll(OneToManyMap<T,U> mapToAdd)` Add all elements of a one2many map to another map |
    | `static OneToManyMap<java.lang.String,java.lang.String>` | OneToManyMap.`invertKeysAndValues(OneToManyMap<java.lang.String,java.lang.String> map)` |
  - ### Uses of OneToManyMap in uk.ac.standrews.utils.main.setutils

    Methods in uk.ac.standrews.utils.main.setutils that return OneToManyMap

    | Modifier and Type | Method and Description |
    | --- | --- |
    |  |  |
    | --- | --- |
    | `OneToManyMap` | SupersetsManager.`getSupersetsIds()` |
  - ### Uses of OneToManyMap in uk.ac.standrews.utils.test.maputils

    Methods in uk.ac.standrews.utils.test.maputils that return OneToManyMap

    | Modifier and Type | Method and Description |
    | --- | --- |
    |  |  |
    | --- | --- |
    | `static OneToManyMap<java.lang.String,java.lang.String>` | OneToManyMapTest.`oneToManyMap1()` |
    | `static OneToManyMap<java.lang.String,java.lang.String>` | OneToManyMapTest.`oneToManyMap2()` |
    | `static OneToManyMap<java.lang.String,java.lang.String>` | OneToManyMapTest.`oneToManyMapEmptyKeyValue()` |

- Overview
- Package
- Class
- Use
- Tree
- Deprecated
- Index
- Help

- Prev
- Next

- Frames
- No Frames

- All Classes
